# Telehealth Intervention to Reduce Sedentary Behavior in Older Adults With Type 2 Diabetes: Development and Feasibility Study

**DOI:** 10.2196/80827

**Published:** 2026-03-26

**Authors:** Xiaoyan Zhang, Dan Yang, Sihan Chen, Lei Wang, Xin Li, Ziyan Wang, Meiqi Meng, Xuejing Li, Hongzhan Jiang, Yufang Hao, Jiayin Luo

**Affiliations:** 1 School of Nursing, Beijing University of Chinese Medicine, Liangxiang High Education Park, Fangshan District, Beijing, 102488, China, 86 18911091028; 2 Department of Vascular Surgery, Beijing Hospital, National Center of Gerontology, Institute of Geriatric Medicine, Chinese Academy of Medical Sciences, Beijing, China; 3 Department of Nursing Administration, Fangshan Hospital Beijing University of Chinese Medicine, Beijing, China; 4 Department of Nursing, Beijing Hospital, National Center of Gerontology, Institute of Geriatric Medicine, Chinese Academy of Medical Sciences, Beijing, China

**Keywords:** intervention mapping, behavior change intervention, sedentary behavior, older adults, mixed methods, telehealth interventions

## Abstract

**Background:**

Sedentary behavior (SB) is a modifiable risk factor for complications in older adults with type 2 diabetes mellitus (T2DM). Despite widespread adoption of digital health platforms, theory-driven telehealth interventions specifically targeting SB reduction remain limited, particularly those incorporating cultural adaptation and behavioral change frameworks.

**Objective:**

This study aims to develop and evaluate the feasibility of a theory-based personalized telehealth intervention to reduce SB in older adults with T2DM in China.

**Methods:**

The intervention was developed over 14 months (January 2022-February 2023) following the intervention mapping and Behavior Change Wheel frameworks. A panel of 19 multidisciplinary experts (90.5% response rate) refined the program through a systematic iterative process. Subsequently, a 7-week quasi-experimental study (pre-post self-controlled design) was conducted to assess feasibility. We recruited 30 community-dwelling older adults with T2DM via WeChat-based convenience sampling. The primary outcome was SB measured by the Measure of Older Adults’ Sedentary Time for Type 2 Diabetes Mellitus questionnaire. Secondary outcomes included cardiovascular risk (blood pressure), glycemic control (fasting blood glucose), Diabetes-Specific Quality of Life, social isolation, BMI, and fall incidence. Pre-post changes from baseline to 7 weeks were statistically evaluated to assess the intervention’s feasibility and preliminary impact.

**Results:**

The intervention comprises 5 components: an eHealth education manual, a motion graphics library, an SMS text messaging library, a WeChat Q&A group, and a material incentive package. These components address “knowledge,” “social support,” and “intention” determinants through “education,” “enablement,” and “incentivisation” functions, respectively. All components used the “service provision” policy and various behavior change techniques. Preliminary feasibility testing (n=31) showed reduced sedentary time by 1.12 hours/day (*P*<.001) and improved social connectivity scores (*P*=.001).

**Conclusions:**

This study demonstrates the feasibility and potential impact of a systematically developed telehealth intervention for reducing SB in older adults with T2DM in China. The integration of intervention mapping with the Behavior Change Wheel provides a replicable framework for developing theory-driven digital health interventions. With significant reductions in sedentary time and improved social connectivity, this culturally adapted approach offers a scalable model for chronic disease self-management in aging populations. The systematic methodology and positive preliminary outcomes support further large-scale evaluation of evidence-based telehealth solutions for behavioral modification in diabetes care.

## Introduction

Type 2 diabetes mellitus (T2DM) represents a global health challenge characterized by insufficient insulin secretion and/or dysfunction [1]. The International Diabetes Federation reported that diabetes prevalence among older adults (aged ≥65 y) reached 19.3% globally in 2020, with projections indicating an increase to 27.6% by 2045 [2]. This trend is particularly pronounced in upper-middle-income countries (UMICs), where age-standardized mortality rates remain substantially elevated [3]. China exemplifies this challenge with a T2DM prevalence of 30.2% among its older population [4]. Given the profound impact, lifestyle modifications offer a potentially cost-effective and sustainable method for improving T2DM outcomes [5], with sedentary behavior (SB) emerging as a critical modifiable risk factor.

SB is defined as any waking behavior characterized by an energy expenditure of 1.5 metabolic equivalents or less while sitting, reclining, or lying [6]. Distinct from physical inactivity, adverse health effects may persist even when recommended activity levels are met [7]. Globally, 67% of adults aged 60 years and older spend over 8.5 hours daily in SB [8], with 69.8% of Chinese older adults with diabetes classified as sedentary [9]. Extensive research confirms SB’s adverse impacts, including increased all-cause mortality [10], cardiovascular disease [11], cancer [12], and metabolic disorders [13-16]. SB represents a significant modifiable risk factor requiring targeted intervention in T2DM management.

While the World Health Organization (WHO) and various countries’ guidelines recommend reducing sedentary time [17-19], promoting physical activity alone proves insufficient [20,21]. Current SB interventions face challenges: moderate activity often replaces light activity rather than sedentary time [22], and recommended activity levels may paradoxically increase compensatory SB [23]. Notably, a 2021 Cochrane systematic review revealed no statistically significant effects of current SB interventions for community-dwelling older adults [24]. As the global chronic disease burden increases, telemedicine emerges as a crucial tool for providing accessible care [25]. While small-scale studies suggest potential efficacy [26], patient-oriented behavioral interventions remain fragmented—a challenge prevalent globally in digitally advancing health care systems [27,28]. There is a pressing need for theory-based, culturally adapted approaches that systematically integrate theoretical frameworks into remote SB intervention design.

Traditional implementation strategy development often relies on the “It Seemed Like a Good Idea at the Time” principle, targeting barriers without theoretical foundation, thereby compromising outcome interpretation and failing to elucidate mechanisms of action [29]. The UK Medical Research Council recommends systematic, theoretically grounded approaches [29]. Implementation science has emerged as a discipline exploring how to systematically integrate evidence-based interventions into routine practice [30-32]. To operationalize these principles, the intervention mapping (IM) framework [33,34] provides a structured, theory-driven approach that constructs logical problem models, identifies behavioral determinants, and matches them with behavioral objectives. Successfully applied in SB reduction interventions across diverse populations [35-38], IM ensures systematic development of theoretically grounded strategies.

**Figure 1.**
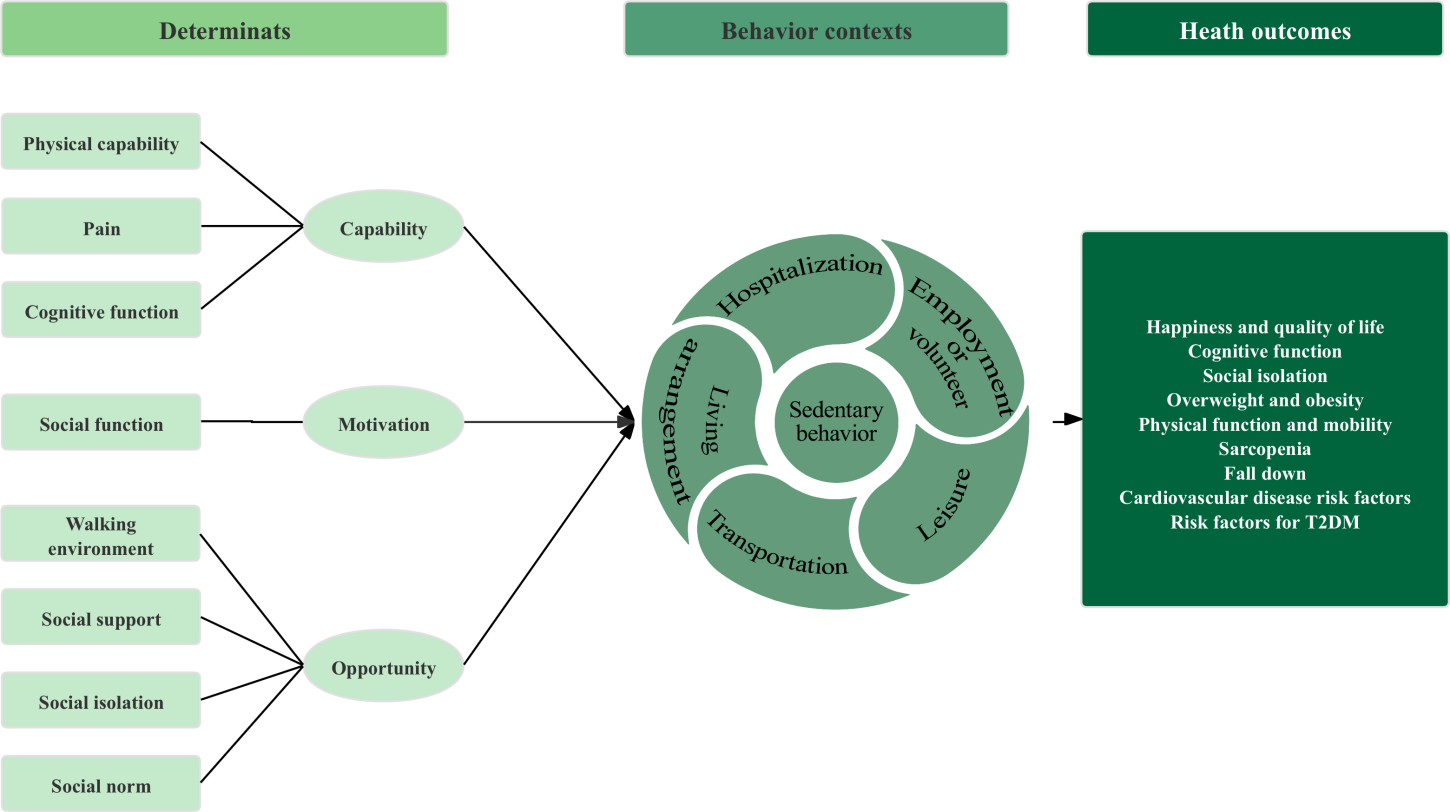
Preliminary conceptual framework of sedentary behavior for older adults with type 2 diabetes mellitus (T2DM). This framework was developed prior to empirical data collection by integrating the capability, opportunity, motivation, and behavior model, the theoretical domains framework, and existing literature. The model depicts a logical flow from potential determinants mapped onto COM-B dimensions on the left, through various behavioral contexts in the center, to potential health outcomes on the right. This initial framework was subsequently validated and refined through 2-phase empirical studies ([39]; X Zhang et al, PhD, unpublished data, August 2025), leading to the finalized logic model presented in Figure 2.

**Figure 2.**
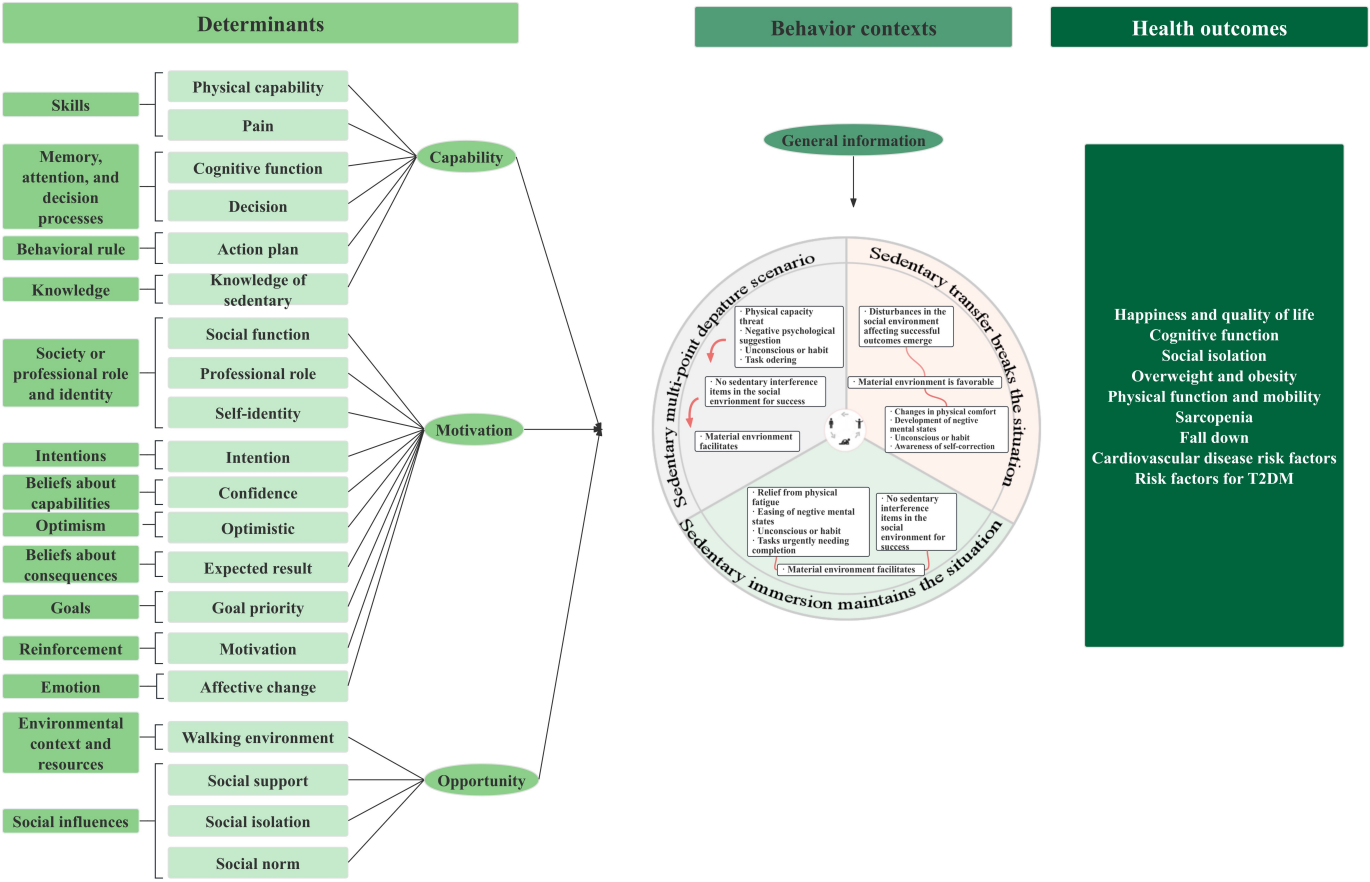
Logical model of sedentary behavior in older adults with type 2 diabetes mellitus (T2DM).

Our foundational work involved completing step 1 of the IM framework through previously published research [39]. This foundational phase used a 2-phase sequential explanatory mixed methods design based on the Behavior Change Wheel (BCW). Initially, we used an established conceptual framework (Figure 1) to conduct a cross-sectional survey, which validated key SB patterns and determinants [39]. Subsequently, qualitative interviews were conducted to enrich the model with patient perspectives and identify contextual influencing factors (X Zhang et al, PhD, unpublished data, August 2025). Through the integration of these findings, we finalized the SB logic model (Figure 2), which systematically delineates 20 modifiable determinants across capability, motivation, and opportunity dimensions, as well as a 3-stage behavioral trajectory: multipoint triggering, immersive maintenance, and transitional breaking contexts. This logic model (Figure 2) serves as the theoretical and empirical foundation for the systematic development and testing of the “Double-S” intervention in this study.

Building on these foundational insights, this study aims to translate identified behavioral determinants into a practical, technology-enabled application. We introduced the theory-driven remote intervention program “Double-S” (stop SB, stand up), designed to specifically address the identified determinants and reduce SB among community-dwelling older adults with T2DM. This research sought to bridge the gap between theoretical frameworks and practical telehealth delivery by addressing the following specific research questions: (1) How can a multicomponent telehealth intervention be systematically developed for older Chinese adults with T2DM using the IM [33,34,40] and BCW [41] frameworks? (2) Is the resulting “Double-S” program feasible and acceptable for this population, and what are its preliminary impacts on behavioral and health-related outcomes?

## Methods

### Study Design

**Figure 3.**
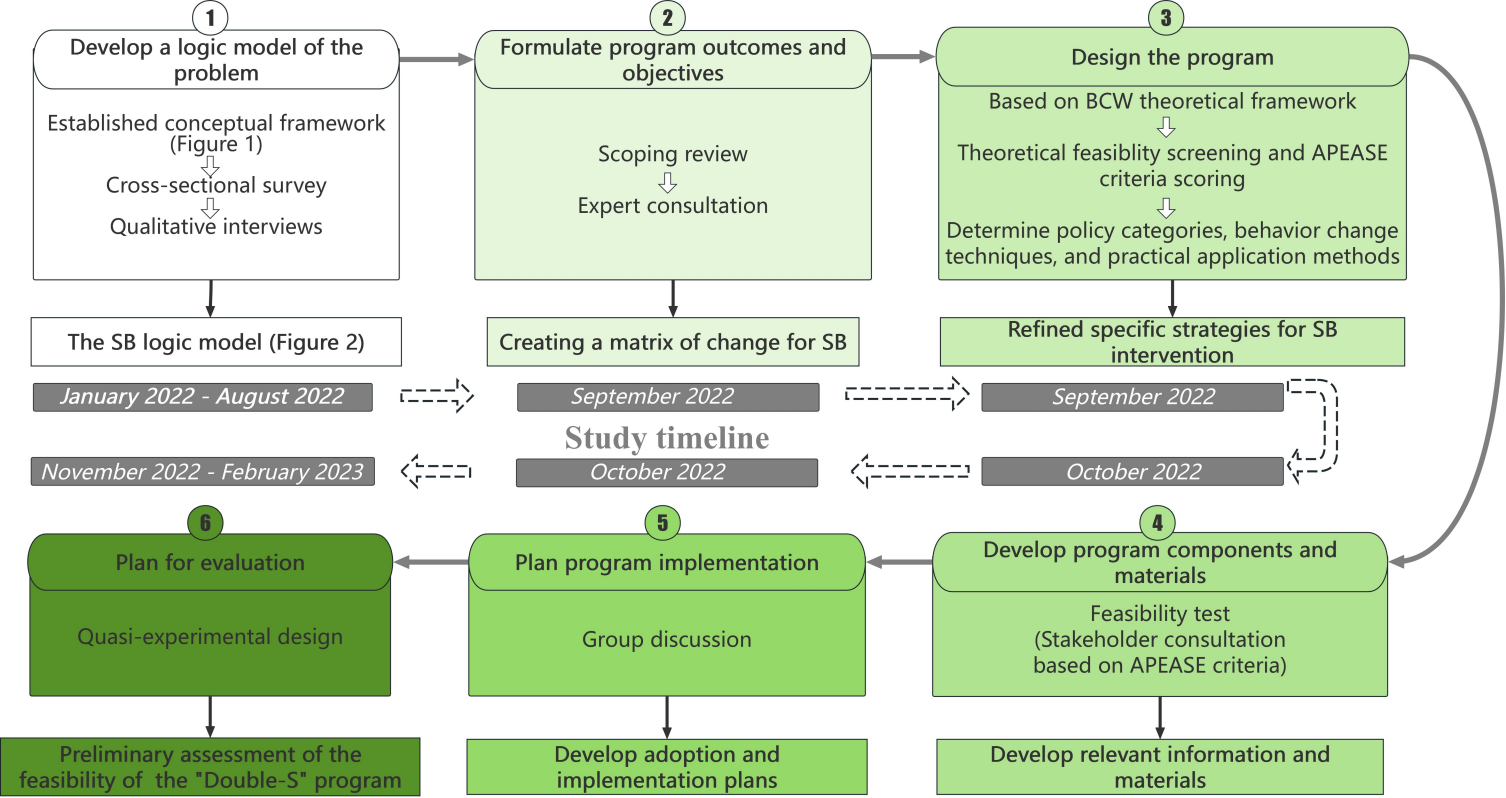
Intervention mapping (IM) process and study timeline for the “Double-S” program. APEASE: Acceptability, Practicability, Effectiveness, Affordability, Safety, Equity; BCW: Behavior Change Wheel; SB: sedentary behavior.

This study used the IM framework to develop and evaluate a theory-based behavior change intervention. The process focused on IM steps 2 through 6, conducted between January 2022 and February 2023, spanning 14 months (Figure 3). Building on the logic model established in step 1 (Figure 2), which was previously completed and published [39], this study focuses on IM steps 2 through 6. A detailed summary of the sampling strategies, recruitment channels, and participant involvement (and the independence of participants) across all stages is provided in Multimedia Appendix 1. IM consists of six iterative steps: (1) completed previously [39], (2) formulating program outcomes and objectives, (3) designing the program, (4) producing program components and materials, (5) planning program implementation, and (6) planning for evaluation. Each step used core processes to identify key literature, apply theory, and collect additional data [40]. Experts were purposively recruited through professional networks and institutional recommendations between January 2022 and February 2022. The research team identified potential participants based on their expertise and extended personal invitations via email, accompanied by detailed program information. The panel comprised experts from various fields: implementation science, evidence-based practice, education, psychology, geriatric nursing, clinical nursing, and diabetes specialist nursing. Eligibility criteria required professionals to hold a relevant postgraduate degree (master’s or higher) or possess over 10 years of specialized work experience. Of the 21 professionals identified and invited, 19 accepted the invitation (90.5% response rate). The recruitment was conducted through the research team’s professional database and academic societies related to geriatric nursing in Beijing. No formal interviews were conducted; selection was based solely on professional credentials and alignment with the program’s objectives.

### Ethical Considerations

The study protocol was approved by the ethics committee of Beijing University of Chinese Medicine (2022BZYLL0505). Digital or written informed consent was obtained from all participants prior to their enrollment. To protect participant privacy, all data were anonymized and deidentified during analysis. All participants (experts, stakeholders, and older adults) participated voluntarily and did not receive financial compensation for their involvement.

### Determining Program Outcomes and Change Objectives

This phase aimed to establish behavioral outcomes for the SB intervention and articulate performance objectives (POs) for each outcome, specifying what participants must learn, do, or change to achieve particular results. The process encompassed three key steps. First, based on preliminary literature research, we defined expected behavioral change outcomes for SB intervention strategies by synthesizing evidence from multiple sources: (1) the Cochrane systematic review [24] examined the effectiveness of interventions to reduce sedentary time in adults with type 2 diabetes, providing evidence on the feasibility and health benefits of reducing daily sedentary time; (2) our scoping review [42] systematically mapped existing evidence on SB interventions in older adults, complementing the disease-specific evidence from the Cochrane review with age-related considerations; and (3) the clinical practice guideline for exercise therapy of T2DM in China (2024 edition) [43] provided specific recommendations on the maximum duration of single sedentary bouts for patients with T2DM. These reviews complemented each other by providing both disease-specific evidence (population with T2DM) and age-specific considerations (older adults), enabling us to establish appropriate behavioral targets for our specific population of older adults with T2DM. For these expected outcomes, we set POs based on the previously developed 3-stage SB trajectory model (specifically, sub-behaviors of the target behavior). These POs specified what the study participants needed to learn, implement, or change to achieve the specific outcomes. Second, selecting important and modifiable behavioral determinants. Guided by the SB logic model, we developed an expert consultation questionnaire to systematically evaluate each determinant. This evaluation was conducted by the intervention strategy development group, consisting of a multidisciplinary panel of 19 experts with a mean professional or research experience of 7.39 (SD 6.94) years. The panel’s expertise spanned various fields, including implementation science, evidence-based practice, education, psychology, geriatric nursing, community nursing, and specialized diabetes nursing. Members of the intervention strategy development group (n=19) independently rated each determinant on 2 dimensions using a 5-point Likert scale (1=“very low” to 5=“very high”): (1) importance—the extent to which the determinant influences SB in older adults, and (2) modifiability—the extent to which the determinant can be changed through intervention strategies. Determinants scoring 3 or more (indicating moderate to high levels) on both dimensions were selected as priority targets for intervention development. This scoring and threshold system was established to ensure that the resulting intervention would focus on determinants with the highest potential for meaningful behavior change and practical feasibility. Third, creating a matrix of change for SB. The intervention strategy group constructed a change matrix based on the screened determinants and established POs. This step aimed to operationalize objectives, enhancing their practicality and feasibility.

### Program Design

#### Framework

This phase aimed to translate change objectives into practical intervention strategies using a theory-driven approach. We adopted the BCW [41] as our overarching framework, as it systematically integrates the Capability, Opportunity, Motivation, and Behavior (COM-B) model, the theoretical domains framework (TDF), and behavior change techniques (BCTs). The COM-B model explains behavior change mechanisms by analyzing the interplay of 3 factors: capability, opportunity, and motivation. Building on COM-B, the TDF provides a more detailed classification of behavioral determinants (Multimedia Appendix 2). BCTs are specific intervention measures designed to modulate behavioral determinants [44]. Based on this theoretical framework and its components, intervention strategies were developed through the following steps.

#### Identifying Intervention Functions

Based on the relationship between determinants identified by the TDF and intervention functions recommended by the BCW (Multimedia Appendix 3), we screened theoretically feasible intervention functions. Subsequently, the intervention development group applied the APEASE (Affordability, Practicability, Effectiveness/Cost-effectiveness, Acceptability, Side-Effects/Safety, Equity) criteria [45] to score the prescreened intervention functions. Functions with the highest scores were selected for strategy design. The APEASE scoring system ranged from 1 to 3, with higher scores indicating greater development potential for the intervention function (Multimedia Appendix 4).

#### Determining Policy Categories, BCTs, and Practical Application Methods

First, we identified theoretically applicable policy categories to support the selected intervention functions, primarily based on the BCW-recommended matching between intervention functions and policy categories (Multimedia Appendix 3). Subsequently, we referred to the results of our previous literature review [42] on SB interventions for older adults and the BCTs recommended in the BCW guide that correspond to specific intervention functions. Group members then determined the BCTs by mapping the selected intervention functions to the specific needs of older adults with T2DM, guided by both the BCW manual and findings from our previous literature review [42]. Finally, considering the identified intervention functions, policy categories, and BCTs, as well as the context and environment of intervention implementation, the intervention development group further refined specific strategies for SB intervention in community-dwelling older adults with T2DM.

### Program Production

This phase aimed to refine the structure of intervention strategies and to draft, test for feasibility, and produce relevant information and materials. The intervention strategy group developed an initial draft of SB intervention strategies for community-dwelling older adults with T2DM, based on previously identified functions, policy categories, and BCTs. Throughout the iterative process, POs, change objectives, and practical strategies were continuously optimized.

To test the feasibility of strategies, a stakeholder consultation method was used. A panel of 9 stakeholders was recruited through purposive sampling, including experts in evidence-based medicine and nursing, specialists in education, clinicians in diabetes care, and patient representatives. These 9 stakeholders were independent of the 19 experts involved in step 2 to ensure an objective external review, although they shared similar eligibility criteria. The consultation was conducted through (eg, individual email-based or face-to-face sessions). Each stakeholder was provided with a detailed description of the proposed intervention and used a custom-designed APEASE scoring sheet to evaluate intervention strategies across 6 dimensions: acceptability, practicability, effectiveness or cost-effectiveness, affordability, side effects or safety, and equity. Each dimension was rated on a 3-point Likert scale. Data were analyzed using descriptive statistical methods.

### Program Implementation Plan

This phase focused on developing adoption and implementation plans in consultation with the intervention development group. Key stakeholders from evidence-based medicine or nursing, education, and diabetes care were engaged to discuss the integration of intervention strategies into existing services, as well as the logistics of delivery (how, when, and by whom). Training guidelines and curriculum plans were developed to equip community older people’s health and diabetes management professionals with the tools to address the primary barriers contributing to SB in older adults with T2DM. In designing remote interventions, we specifically considered the resource constraints and technological infrastructure challenges common in UMICs. We opted for low-cost, easily implementable remote intervention solutions that do not require expensive equipment or complex technical support.

### Evaluation Plan

This phase aimed to preliminarily assess the feasibility of the “Double-S” program for community-dwelling older adults with T2DM. A quasi-experimental design (pre-post self-controlled study) was used (registration: ER20220111).

The study population comprised community-dwelling older adults with T2DM. A convenience sampling method was used to recruit participants via WeChat in November 2022. Recruitment flyers were posted in 3 purposively selected WeChat groups of community-dwelling older adults and shared through the “Moments” functions of community health workers. Interested individuals contacted the research team directly via WeChat or phone. The researchers then screened these potential participants against the inclusion criteria and explained the study’s purpose. Eligible candidates who provided informed consent were then purposively selected to ensure diversity in age and health status and formally enrolled for interviews. Inclusion criteria were: aged 60 years and older, diagnosed with T2DM, permanent community residents, ability to use WeChat on smartphones, and voluntary participation. Exclusion criteria included communication disorders, mental disorders, physical disabilities preventing standing, and comorbid severe illnesses. No formal sample size calculation was conducted due to the exploratory nature of this feasibility study; a target sample of 30 patients was set.

The “Double-S” program was delivered over a 7-week period using a WeChat-based telehealth approach. The intervention was facilitated by a trained research assistant and consisted of four primary components as follows:

- Digital education: an eHealth education manual, designed as a cloud-based document, was provided to participants for real-time access.
- Behavioral prompts: standardized reminders were selected from a predeveloped prompt library and sent to the WeChat group to encourage participants to reduce and break sedentary bouts.
- Visual learning: motion graphics (MG) animations were distributed through the WeChat group and a synchronized public video channel to demonstrate sedentary-breaking exercises.
- Interactive support: real-time Q&A sessions were conducted within the WeChat group to address participant concerns and provide peer support.

Participants underwent structured assessments at 2 specific time points: baseline (T0; week 0) and postintervention (T1; immediately following the completion of week 7). To ensure data accuracy and efficiency, all assessments were conducted online via the “Wenjuanxing” professional survey platform (similar to SurveyMonkey). The research team provided remote guidance to participants who required assistance in navigating the electronic questionnaires.

The intervention implementation adhered to the plan formulated in step 5, with assessment focusing on primary and secondary outcome measures. The primary outcome, SB, was measured using the Measure of Older Adults’ Sedentary Time for T2DM Patients questionnaire [39]. Secondary outcomes encompassed a comprehensive range of health and psychosocial factors: cardiovascular disease risk (assessed by the most recent morning blood pressure readings), T2DM risk factors (evaluated using the most recent fasting blood glucose levels), quality of life (measured with the Diabetes-Specific Quality of Life scale) [46], social isolation (assessed using the Chinese version of the Lubben Social Network Scale-6 [LSNS-6]) [47,48], overweight and obesity (determined by BMI), and fall incidence (recorded as the number of individuals experiencing falls and their consequences within the past week).

Data were analyzed by SPSS Statistics v25.0 (IBM Corp). Descriptive statistics were used for continuous variables. Paired 2-tailed *t* tests or Wilcoxon signed-rank tests were used to compare preintervention and postintervention differences, depending on data distribution. The McNemar test was used for paired categorical variables. The significance level for all statistical tests was set at .05.

## Results

### Program Outcomes and Change Objectives

#### Behavioral Change Outcomes and POs

Based on the previously established logic model ([39]; X Zhang et al, PhD, unpublished data, August 2025), the intervention strategy group developed the following behavioral change outcome and POs. First, older adults with T2DM should reduce their average sedentary time by 1 hour per day (based on evidence synthesis from Cochrane systematic review [24] and our scoping review [42]), with a recommendation that single bouts of SB not exceed 30 minutes (based on the *Guideline for Exercise Therapy of T2DM in China, 2024 Edition* [43]). Second, to achieve the expected behavioral outcome, 4 POs were established based on the 3-stage trajectory of SB (Multimedia Appendix 5) as follows:

- PO1: patients should comprehend the risks and consequences of SB (prebehavior).
- PO2: patients select appropriate strategies to address SB (during behavior).
- PO3: patients implement reminders and interruptions during sedentary periods (during behavior).
- PO4: patients engage in appropriate physical activity to alleviate discomfort from SB (postbehavior).

#### Selection of Important and Modifiable Determinants of SB

Following the evaluation by the multidisciplinary panel described in the “Program Production” section, the assessment results (Table 1) identified knowledge, social influences, and intention as the priority TDF domains meeting both importance and modifiability criteria. These identified determinants were mapped directly to the TDF framework during the analysis. Further analysis within the social influences domain revealed that it encompassed social support, social isolation, and social norms. Considering feasibility constraints, social support was selected as the representative factor for social influence.

**Table 1.** Degree of support, importance, and modifiability scores for the determinants of sedentary behavior.

TDF^a^ domains (behavioral determinants)	Literature review [49]	Cross-sectional study [39]	Qualitative research^b^	Importance, mean (SD)	Modifiability, mean (SD)
Knowledge	N^c^	N	Y^d^	4.00 (0.79)	4.37 (0.81)
Memory, attention, and decision processes	Y	Y	Y	4.74 (0.55)	2.68 (0.92)
Behavioral regulation	N	N	Y	4.58 (0.75)	2.84 (0.74)
Skills	N	Y	Y	4.00 (0.79)	2.79 (0.77)
Environmental context and resources	N	Y	Y	3.95 (1.05)	2.53 (1.09)
Social influences	N	Y	Y	3.63 (0.98)	3.47 (0.60)
Society or professional roles and identity	N	Y	Y	3.84 (0.87)	2.74 (0.85)
Intentions	N	N	Y	4.26 (0.91)	3.16 (0.49)
Beliefs about capabilities	N	N	Y	4.16 (0.67)	2.95 (0.76)
Optimism	N	N	Y	3.58 (1.09)	2.79 (0.83)
Beliefs about consequences	N	N	Y	3.47 (1.14)	2.74 (0.64)
Goals	N	N	Y	3.58 (1.04)	2.32 (0.46)
Reinforcement	N	N	Y	3.47 (0.60)	2.89 (0.64)
Emotion	N	N	Y	3.21 (0.77)	2.47 (0.60)

a TDF: theoretical domains framework.

b X Zhang et al, PhD, unpublished data, August 2025.

c N: not yet discovered.

d Y: support.

#### Change Matrix for SB

Based on the established POs and the selected determinants, specific change objectives were formulated to create a comprehensive change matrix, as shown in Table 2.

**Table 2.** Matrix of change objectives for sedentary behavior reduction: linking performance objectives (POs) with theoretical determinants.

PO	Determinants		
	Knowledge	Social support	Intentions
PO1: the patient should comprehend the risks and consequences of SB (before behavior).	The patient should comprehend the hazards of SB^a^ (current situation, consequences, and its relationship with T2DM^b^).	Researchers or family members provide social support to the patient regarding PO1.	The patient expresses an intention to reduce SB.
PO2: the patient chooses appropriate strategies to cope with SB (during behavior).	The patient should comprehend strategies to counteract SB, such as reminders and behavior reversal.	Researchers or family members provide social support to the patient regarding PO2.	The patient expresses an intention to adopt appropriate coping strategies.
PO3: The patient is reminded and interrupted during SB (during behavior).	Family members should comprehend the hazards of SB (current situation, consequences, and its relationship with T2DM).	Researchers or family members provide social support to the patient regarding PO3.	Family members express an intention to monitor and remind the patient.
PO4：the patient engages in appropriate physical activities to alleviate discomfort from SB (after behavior).	The patient should comprehend how to engage in physical activity.	Researchers or family members provide social support to the patient regarding PO4.	The patient expresses an intention to engage in physical activity.

a SB: sedentary behavior.

b T2DM: type 2 diabetes mellitus.

### Program Design

#### Selection of Intervention Functions

Intervention functions were evaluated using the APEASE criteria, with results presented in Multimedia Appendix 6. The BCW framework was used to align intervention functions with determinants: (1) knowledge: education (mean total score of 16.89, SD 1.10), (2) intentions: incentivization (mean highest score of 15.21, SD 1.08), and (3) social support: enablement (mean highest score of 15.10, SD 1.33).

#### Determination of Policy Categories, BCTs, and Practical Applications

The theoretical constructs were systematically translated into practical intervention strategies through integration of the following components: behavioral change outcomes, POs, determinants, change matrix, intervention functions, and policy categories. The resulting practical applications are comprehensively outlined in Multimedia Appendix 7.

### Intervention Production

#### Draft Intervention Strategies

Through 2 rounds of expert panel discussions (n=19), the group determined that simple SMS text messaging reminders and social media–based support groups were particularly effective in reducing SB. These low-cost, highly acceptable intervention methods may be applicable in other resource-limited settings. Following two rounds of expert panel discussions involving 19 intervention group specialists, a comprehensive intervention strategy for SB in the community-dwelling older adults with T2DM was formulated. The strategy comprised 5 key components: eHealth education manual, MG animation library, SMS text messaging library, WeChat Q&A group, and material incentive package. The eHealth education manual, MG animation library, and SMS text messaging library targeted the “knowledge” determinant of SB. The WeChat Q&A group primarily addressed the “social support” determinant, while the material incentive package focused on the “intentions” determinant. Full descriptions of these intervention strategies are provided in Multimedia Appendix 8.

#### Feasibility Assessment of Intervention Strategies

Eight multidomain stakeholders (mean age of 44.75, SD 15.56 y) participated in the feasibility assessment, including 2 implementation science and evidence-based nursing or medicine stakeholders, 2 education stakeholders, 2 diabetes clinical nursing stakeholders, and 2 patient representatives. APEASE scoring results are presented in Multimedia Appendix 9. The eHealth education manual, MG animation library, SMS text messaging library, and material incentive package all achieved mean scores of 3.00 (SD 0.00), while the WeChat Q&A group scored 2.88 (SD 0.35).

#### Program Implementation Plan

Specific POs for intervention implementation are outlined in Table 3. These objectives were established by the research team based on the behavioral determinants identified in the logic model (Table 2). To achieve the adoption and implementation change objectives, the intervention design group selected a series of BCTs grounded in theory and integrated them with the 5 intervention components (Multimedia Appendix 10): eHealth education manual, MG animation library, SMS text messaging library, WeChat Q&A group, and material incentive package.

**Table 3.** Adoption and implementation outcomes and performance objectives (POs) for the intervention program.

PO	Context
PO1	The patient should comprehend the hazards of SB^a^ (before behavior).
PO2	The patient should comprehend strategies to counteract SB, such as reminders and behavior reversal.
PO3	Family members should comprehend the hazards of SB (current situation, consequences, and its relationship with T2DM^b^).
PO4	The patient should comprehend how to engage in physical activity.

a SB: sedentary behavior.

b T2DM: type 2 diabetes mellitus.

### Evaluation Results

A total of 31 community-dwelling older adults with T2DM (mean age 66.35, SD 3.94 years) completed the 7-week intervention. All 31 participants successfully completed both the baseline (T0) and postintervention (T1) assessments. Detailed participant characteristics are provided in Multimedia Appendix 11.

Following the implementation of the intervention, significant reductions in SB were observed among participants. Total sedentary time decreased by 1.12 hours per day compared to preintervention levels (*P*<.001). Notably, screen-based sedentary time showed a significant reduction of 0.33 hours per day (*P*=.01). However, no statistically significant changes were observed in sedentary time related to reading (*P*=.71), socializing (*P*=.87), transportation (*P*=.32), hobbies (*P*=.85), or other activities (*P*=.56).

The intervention also yielded improvements in social network scores as measured by the LSNS-6. Post-intervention LSNS-6 scores showed significant improvement (*P*<.001), with the family dimension demonstrating marked enhancement (*P*<.001). The friend dimension, however, did not show statistically significant changes (*P*=.46).

For participants with T2DM, no statistically significant changes were observed in morning blood pressure, fasting blood glucose, Diabetes-Specific Quality of Life scores, or BMI. No fall incidents were reported either before or during the intervention period.

## Discussion

### Principal Findings

This study aimed to develop and evaluate a theory-driven remote intervention strategy, “Double-S,” to reduce SB among older adults with T2DM in China. Our findings suggest that the systematic application of the IM framework is a feasible approach for developing targeted behavioral interventions. To our knowledge, this research represents one of the first theory-driven and evidence-based remote intervention strategies specifically targeting SB in older Chinese adults with T2DM in community settings. By integrating theoretical frameworks and addressing the specific needs of a rapidly aging population in a digitally evolving environment, this work contributes to the growing body of knowledge on culturally adapted, evidence-based interventions for chronic disease management in diverse global settings. Specifically, the intervention led to a promising reduction in total sedentary time by 1.12 hours per day and screen-based sedentary time by 0.33 hours per day. Furthermore, it significantly enhanced social network scores within the family dimension. However, these results should be interpreted as preliminary indicators of the program’s potential rather than definitive proof of efficacy, given the feasibility nature and limited sample size of this pilot evaluation.

### Comparison With Prior Work and Implications

The core advantage of IM lies in its ability to explicitly link intervention strategies with theory, supporting hypothesis testing and deepening our understanding of causal mechanisms [33,50]. By addressing the gap in theory-grounded, culturally adapted digital health tools, our study offers a novel design contribution to the chronic disease management market in China and other similar settings. In diverse health care settings, IM is crucial for developing systematic approaches to use available resources and translate research findings into routine practice. Building on the BCW theory, this study constructed a problem logic model for SB among older adults with T2DM. The model integrates determinants (capability, motivation, and opportunity), the Chinese community context, the 3-stage SB trajectory, and health outcomes. By addressing this gap, our study offers new perspectives on improving behavior change interventions in diverse health care environments.

While IM has been widely applied in Western health care contexts [51-53], our research explores its applicability in Asian settings. Previous reviews [42] indicated that Western interventions predominantly target individual psychological aspects through face-to-face counseling or wearable devices.

However, SB is a habitual, unconscious behavior deeply influenced by cultural and environmental factors. The perception of SB and social norms varies across cultural contexts, affecting not only individual behavior but also health system design. Consequently, Western strategies need to be re-examined and adapted for Asian health care settings to ensure their effectiveness.

In community-based settings like China, high-cost individualized interventions pose challenges due to population aging and diabetes pressures [54]. Thus, our research shifted focus towards social and policy-level intervention strategies. Initial findings suggest that older adults in China often obtain health information through public media, aligning with current science popularization policies. The high feasibility and acceptability scores from both multidisciplinary stakeholders and older users highlight significant opportunities for scaling up the “Double-S” program.

The widespread use of smartphones offers new channels for rapid health information dissemination. Remote interventions combined with digital tools provide advantages such as broad coverage and cost-effectiveness. Given the deep integration of digital devices into daily life, leveraging digital means for diabetes management has become increasingly necessary [55].

Beyond testing under controlled conditions, the preliminary evaluation results from both stakeholder and user perspectives provide a strong foundation for future implementation testing. This includes exploring how the “Double-S” program can be integrated into routine community health care systems for large-scale application. However, while the “Double-S” proved feasible under controlled conditions, real-world implementation may be constrained by structural barriers. This finding highlights potential challenges in transitioning from experimental settings to practical applications.

Digital infrastructure in rural areas and technological adaptability among older populations generally lag behind urban areas [56]. This can subject beneficiaries to “double exclusion” regarding technology access and service availability. Policymakers should prioritize bridging the gap between “technology popularization” and “institutional adaptation” rather than merely hardware upgrades.

Regarding effectiveness, the results showed significant improvements in social isolation but limited impact on physiological indicators like BMI or blood glucose. This aligns with findings from Ferguson et al [57] and Chastin et al [24], who indicated that metabolic improvements from SB reduction are typically modest and nonsignificant in the short term.

These findings reveal the dual nature of remote interventions: while cost-effective in enhancing social support networks, they may face challenges in reversing long-term metabolic disorders quickly. Heterogeneity in intervention intensity and duration likely compromises cross-study comparability, suggesting that determining core parameters within specific cultural contexts is crucial.

### Limitations

This study has several limitations that should be acknowledged. First, the feasibility study used a convenience sampling method with a small sample size (N=31), which may limit the generalizability of the findings to the broader population with T2DM in China. Importantly, as this was a feasibility study, it lacked the statistical power to definitively assess clinical effectiveness; thus, the observed reductions in sedentary time should be viewed as preliminary. Second, the 7-week intervention period was relatively short, which likely explains the lack of significant changes in physiological and metabolic outcomes. Third, data collection relied on self-reported questionnaires, which are subject to recall bias. Future research should use randomized controlled trials with longer follow-up periods and objective measures of SB to further validate these findings.

### Conclusions

The “Double-S” program demonstrates that theory-driven telehealth interventions can be successfully developed and adapted to specific cultural and technological contexts to address the public health challenge of SB. Beyond the immediate reduction in sedentary time, this research highlights the critical role of social media ecosystems and family-based social support in managing chronic diseases among older adults. Rather than providing definitive evidence of efficacy, this study offers a scalable design model and a validated methodological foundation for health care systems in UMICs. Successfully scaling such digital health solutions requires moving beyond hardware deployment toward institutional adaptation and the development of localized, culturally sensitive outcome sets. The positive outcomes from this pilot phase support the progression toward large-scale implementation trials to further validate the program’s impact.

## Supplementary material

10.2196/80827 Multimedia Appendix 1 Detailed recruitment and participant flow.

10.2196/80827 Multimedia Appendix 2 The matching relationship between the determinants identified by theoretical domains framework and the capability, opportunity, motivation, and behavior components.

10.2196/80827 Multimedia Appendix 3 Mapping relationships between behavioral determinants, intervention functions, and policy categories.

10.2196/80827 Multimedia Appendix 4 APEASE (Affordability, Practicability, Effectiveness/Cost-effectiveness, Acceptability, Side-Effects/Safety, Equity) criteria.

10.2196/80827 Multimedia Appendix 5 Three-stage trajectory of sedentary behavior.

10.2196/80827 Multimedia Appendix 6 Intervention function APEASE (Affordability, Practicability, Effectiveness/Cost-effectiveness, Acceptability, Side-Effects/Safety, Equity) score.

10.2196/80827 Multimedia Appendix 7 The practical application of intervention strategies converts results.

10.2196/80827 Multimedia Appendix 8 “Double-S” intervention strategy for older adult patients with type 2 diabetes.

10.2196/80827 Multimedia Appendix 9 “Double-S” program APEASE (Affordability, Practicability, Effectiveness/Cost-effectiveness, Acceptability, Side-Effects/Safety, Equity) score results.

10.2196/80827 Multimedia Appendix 10 The 5 components of the “Double-S” Plan.

10.2196/80827 Multimedia Appendix 11 General patient information (N=31).

## Abbreviations

APEASE: Affordability, Practicability, Effectiveness/Cost-effectiveness, Acceptability, Side Effects/Safety, Equity
BCT: behavior change technique
BCW: Behavior Change Wheel
COM-B: capability, opportunity, motivation, and behavior
IM: intervention mapping
LSNS-6: Lubben Social Network Scale-6
MG: motion graphics
PO: performance objective
SB: sedentary behavior
T2DM: type 2 diabetes mellitus
TDF: theoretical domains framework
UMIC: upper-middle-income country
WHO: World Health Organization
