# Supplementary material for: Telehealth Intervention to Reduce Sedentary Behavior in Older Adults With Type 2 Diabetes: Development and Feasibility Study
Source: J Med Internet Res. 2026 Mar 26;28:e80827. doi: 10.2196/80827 (PMC13020683; doi:10.2196/80827)
Supplement: Multimedia Appendix 1 [file jmir-v28-e80827-s001.docx]

**Appendix 1: Detailed Recruitment and Participant Flow**

**Table 1. Detailed Overview of Sampling and Participant Involvement**

| **Development Stage** | **Target Population** | **Sampling Strategy** | **Recruitment Channels & Procedures** | **Sample Size (N)** | **Participant Overlap** |
| --- | --- | --- | --- | --- | --- |
| **Stage 2: formulating program outcomes and objectives** | Multidisciplinary Experts | **Purposive Sampling:** Based on expertise in geriatrics, nursing, and psychology. | Recruited via the research team's professional network and institutional recommendations in Beijing. Formal invitations sent via email. | 19 | None (First-stage participants) |
| **Stage 4: producing program components and materials** | Stakeholders (Clinicians & Patient Reps) | **Purposive Sampling:** To ensure diversity in clinical and lived experience. | Identified through community health centers and specialist diabetes clinics. Conducted via individual face-to-face or email sessions. | 9 | **No overlap** with Step 2 experts to ensure objective feedback. |
| **Stage 6: planning for evaluation** | Older adults with T2DM | **Convenience Sampling:** For feasibility and accessibility. | Recruitment posters shared in 3 specific community WeChat groups; health workers shared the flyer in their digital 'Moments'. | 31 | **New participants**; did not participate in previous stages. |
