# Supplementary material for: Telehealth Intervention to Reduce Sedentary Behavior in Older Adults With Type 2 Diabetes: Development and Feasibility Study
Source: J Med Internet Res. 2026 Mar 26;28:e80827. doi: 10.2196/80827 (PMC13020683; doi:10.2196/80827)
Supplement: Multimedia Appendix 2 [file jmir-v28-e80827-s002.docx]

**Appendix 2：The matching relationship between the determinants identified by TDF and the COM-B components**

| COM-B components | | TDF domains | Definition | Theoretical concepts portrayed in each domain |
| --- | --- | --- | --- | --- |
| Capability | Psychological capability | Knowledge | An awareness of the existence of something | Knowledge (including knowledge of condition/scientific rationale), procedural knowledge, knowledge of task environment |
|  |  | Memory, attention and decision processes | The ability to retain information, focus selectively on aspects of the environment and choose between two or more alternatives | Memory, attention, attention control, decision- making, cognitive overload or tiredness |
|  |  | behavioral regulation | Anything aimed at managing or changing objectively observed or measured actions | Self-monitoring, breaking habit, action planning |
|  | Physical capability | Skills | An ability or proficiency acquired through practice | Skills, skills development, competence, ability, practice, skill assessment |
| Motivation | Reflective motivation | Social/professional role and identity | A coherent set of behaviors and displayed personal qualities of an individual in a social or work setting | Professional identity, professional role, social identity, identity, professional boundaries, professional confidence, group identity, leadership, organizational commitment |
|  |  | Beliefs about  capabilities | Acceptance of the truth, reality, or validity about an ability, talent, or facility that a person can put to constructive use | Self-confidence, perceived competence, self-efficacy, perceived behavioral control, beliefs, self-esteem, empowerment, professional confidence |
|  |  | Optimism | The confidence that things will happen for the best or that desired goals will be attained | Optimism, pessimism, unrealistic optimism, identity |
|  |  | Beliefs about  consequences | Acceptance of the truth, reality, or validity about outcomes of a behavior in a given situation | Beliefs, outcome expectancies, characteristics of outcome expectancies, anticipated regret, consequents |
|  |  | Intentions | A conscious decision to perform a behavior or a resolve to act in a certain way | Stability of intentions, stages of change model, transtheoretical model and stages of change |
|  |  | Goals | Mental representations of outcomes or end states that an individual wants to achieve | Goals (distal/proximal), goal priority, goal/target-setting, goals (autonomous/controlled), action planning, implementation intention |
|  | Automatic motivation | Reinforcement | Increasing the probability of a response by arranging a dependent relationship, or contingency, between the response and a given stimulus | Rewards (proximal/distal, valued/not valued, probable/improbable), incentives, punishment, consequents, reinforcement, contingencies, sanctions |
|  |  | Emotion | A complex reaction pattern, involving experiential, behavioral, and physiological elements, by which the individual attempts to deal with a personally significant matter or event | Fear, anxiety, affect, tress, depression, positive/negative affect, burnout |
| Opportunity | Physical opportunity | Environmental context and resources | Any circumstance of a person’s situation or environment that discourages or encourages the development of skills and abilities, independence, social competence, and adaptive behavior | Environmental stressors, resources/material resources, organizational culture/climate, salient events/critical incidents, person environment interaction, barriers and facilitators |
|  | Social opportunity | Social influences | Those interpersonal processes that can cause individuals to change their thoughts, feelings, or behaviors | Social pressure, social norms, group conformity, social comparisons, group norms, social support, power, intergroup conflict, alienation, group identity, modelling |
