# Supplementary material for: Telehealth Intervention to Reduce Sedentary Behavior in Older Adults With Type 2 Diabetes: Development and Feasibility Study
Source: J Med Internet Res. 2026 Mar 26;28:e80827. doi: 10.2196/80827 (PMC13020683; doi:10.2196/80827)
Supplement: Multimedia Appendix 3 [file jmir-v28-e80827-s003.docx]

**Appendix 3: Mapping relationships between behavioral determinants, intervention functions, and policy categories**

**Appendix 3-1:Matching relationship between determinants and intervention function**

| COM-B | TDF | BCW middle ring：Intervention function | | | | | | | | |
| --- | --- | --- | --- | --- | --- | --- | --- | --- | --- | --- |
|  |  | Education | Persuasion | Incentivization | Coercion | Training | Restriction | Environmental restructuring | Modelling | Enablement |
| Physical capability | Skills |  |  |  |  | √ |  |  |  |  |
| Psychological capability | Knowledge | √ |  |  |  |  |  |  |  |  |
|  | Memory, attention, and decision processes |  |  |  |  | √ |  | √ |  | √ |
|  | behavioral regulation | √ |  |  |  | √ |  |  | √ | √ |
| Reflective motivation | Social/professional role and identity | √ | √ |  |  |  |  |  | √ |  |
|  | Intention | √ | √ | √ | √ |  |  |  | √ |  |
|  | Beliefs about  capabilities | √ | √ |  |  |  |  |  | √ | √ |
|  | Optimism | √ | √ |  |  |  |  |  | √ | √ |
|  | Beliefs about  consequences | √ | √ |  |  |  |  |  | √ |  |
|  | Goals | √ | √ | √ | √ |  |  |  | √ | √ |
| Automatic motivation | Reinforcement |  |  | √ | √ | √ |  | √ |  |  |
|  | Emotion |  | √ | √ | √ |  |  |  | √ | √ |
| Physical opportunity | Environmental  context and  resources |  |  |  |  | √ | √ | √ |  | √ |
| Social opportunity | Social influences |  |  |  |  |  | √ | √ | √ | √ |

**Appendix 3-2:Matching relationship between intervention function and policy category**

|  |  | BCW middle ring：Intervention function | | | | | | | | |
| --- | --- | --- | --- | --- | --- | --- | --- | --- | --- | --- |
|  |  | Education | Persuasion | Incentivization | Coercion | Training | Restriction | Environmental restructuring | Modelling | Enablement |
| Outer ring: Policy category | Communication/  marketing | √ | √ | √ | √ |  |  |  | √ |  |
|  | Guidelines | √ | √ | √ | √ | √ | √ | √ |  | √ |
|  | Fiscal measures |  |  | √ | √ | √ |  | √ |  | √ |
|  | Legislation | √ | √ | √ | √ | √ | √ | √ |  | √ |
|  | Regulation | √ | √ | √ | √ | √ | √ | √ |  | √ |
|  | Environmental/social planning |  |  |  |  |  |  | √ |  | √ |
|  | Service provision | √ | √ | √ | √ | √ |  |  | √ | √ |
