# Supplementary material for: Telehealth Intervention to Reduce Sedentary Behavior in Older Adults With Type 2 Diabetes: Development and Feasibility Study
Source: J Med Internet Res. 2026 Mar 26;28:e80827. doi: 10.2196/80827 (PMC13020683; doi:10.2196/80827)
Supplement: Multimedia Appendix 5 [file jmir-v28-e80827-s005.docx]

**Appendix 5：Three-stage trajectory of sedentary behavior**


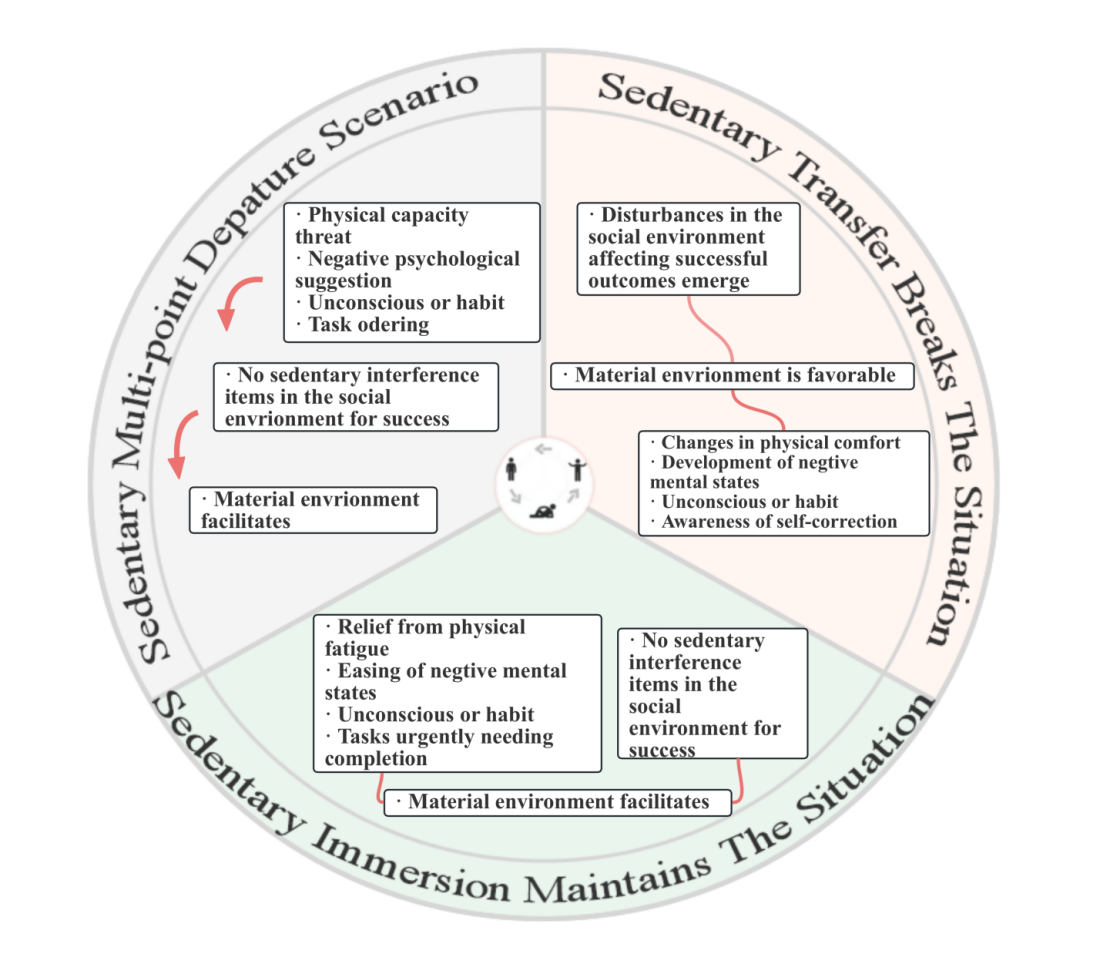


| **Topic** | **Coding** | **Details** | ****Quotation**** |
| --- | --- | --- | --- |
| Sedentary multi-point departure scenario | Physical capacity threat | Elderly T2DM patients often show a decline in physical ability due to age, disease and other factors. When pain, fatigue and other conditions occur, elderly T2DM patients will choose to sit for a long time. In addition, when there are more daily tasks, some patients said that as long as they have time, they will choose to sit for a long time to relieve fatigue. Therefore, the threat of physical ability is the trigger for sedentary situations. | N22: "Sometimes when I'm tired, I like to sit down. Sitting helps to relieve fatigue, and it feels very comfortable."  N14: "It's just exhaustion, there's so much to do... you wouldn't understand being a housewife. There's never-ending work at home. If you want to get things done, you can't sit still for a day... Whenever I have some free time, I just look at my phone... I want to lie down... lying down is more comfortable than sitting." |
|  | Negative psychological suggestion | Sedentary behaviors may also be triggered when older people with T2DM experience negative emotions, such as negative emotions due to the death of a partner, or concerns about physical activity. | N4: "Just walking a bit makes me feel uneasy."  N6: "My husband passed away last December, and before that, I didn’t have this much time to sit. Now that he’s gone, I’m left with just myself. For a while, I was in a bad mood and felt very weak, so I’ve been sitting more, just spending more time sitting during this period." |
|  | Task ordering | The need to complete specific tasks is also a common situation for elderly T2DM patients to trigger sedentary behavior, such as watching an episode of a TV series, reading a wechat article, completing work tasks within a specific time, and so on. When these tasks require a certain amount of continuous time, sedentary behavior often occurs for continuous periods of time. The instructions here may be issued by oneself, or by external individuals, organizations, and groups. | N22: "Since I retired and have nothing to do, playing mahjong requires sitting down. You can't play mahjong while standing."  N7: "For example, if the boss assigns a task, I need 40 minutes or an hour to complete it... or if the boss suddenly gives an urgent task, something like that..." |
|  | Unconsciousness or habit | In addition to mind-body sensations and the triggering of task instructions, one situation that often arises is unconscious or habitual sedentary behavior. Elderly T2DM patients do not think much, and in a specific environment, sedentary behavior occurs spontaneously. This is also the context in which most sedentary behavior occurs in everyday life. | N2: "Habit becomes second nature."  N4: "This is my habit of playing cards, sitting for long periods of time."  N5: "I just watch TV there, and sometimes you don't even realize it."  N10: "Habitual."  N11: "Because if you spend a lot of time sitting, you’ll easily develop a habit." |
|  | No sedentary interference items in the social environment for success. | When sedentary behavior is triggered by physical or mental sensations, task instructions, or unconscious/habitual behaviors, another necessary situation for elderly T2DM patients to develop sedentary behavior is the social environment in which sedentary behavior does not interfere with victory. Society without sedentary interference with victory was divided into two conditions: competition without sedentary behavior at all and competition failure with competitive behavior. When both of these conditions occur, the patient ultimately makes the decision to be sedentary. | N1: "There are a lot of programs I like in the evening, mainly on Channel 4, like 'Asia Today,' 'Focus Today,' 'Cross-Strait News,' and so on. Additionally, there are several other news channels, like those from Shenzhen, Fujian, and Eastern China. So most of my evening time is taken up by these programs." (In other words, the evening programs win the competition for my attention, leading to prolonged sitting.) |
|  | Material environment facilitates | When physical and mental sensations, task instructions or unconscious/habitual trigger sedentary behavior, and there is no sedentary interference with victory items, elderly T2DM patients also need a certain physical environment basis for sedentary behavior, that is, there is a basis for sitting for a long time, such as sofa, stool, chair, etc. In addition, along with the occurrence of sedentary behavior, often appear items include: mobile phones, computers, TV, mahjong, cards, chess, tea cups, etc. Regular sedentary activities include: watching TV, chatting on wechat, scrolling Tiktok, watching news, playing mahjong, playing cards, drinking tea, etc | N1: "After dinner, I mostly just watch TV."  N2: "It's rare that I'm not sitting down. During work hours, I have two pots of tea: first, I go to the office, refill my cup, and then when the client arrives, we have another pot of tea together. In the afternoon, it’s the same—two pots of tea a day."  N6: "Otherwise, I just sit and watch TV. Recently, I’ve been feeling weak all over, so I lie down to watch or sit on the couch to watch." |
| Sedentary immersion maintains the situation | Relief from physical fatigue | When elderly T2DM patients choose to sit for a long time because of the threat of physical ability, it can often be alleviated to a certain extent, and this immediate effect has become the reason why elderly people are willing and like to sit for a long time. | N4: "It feels like my legs have changed, they’re all twisted... I can’t walk for too long, I have to sit down after a while."  N22: "Sometimes when I’m tired, I like to sit down. Sitting helps to relieve fatigue, and it feels really comfortable." |
|  | Easing of negative mental states | When elderly T2DM patients choose to sit for a long time because of negative psychology, their negative emotions can be relieved and transferred to a certain extent. Especially when sitting for a long time while watching TV, scrolling Tiktok and other activities can bring entertainment and temporary perspective shift. When describing a sedentary situation, positive words such as "comfortable, good spirits, like" often appear. | N10: "There's no choice, when it's time to play cards, I feel full of energy."  N11: "It's the weekend, so I just want to relax and stay indoors."  N3: "Sometimes I just sit there, look at my phone, and do some financial planning. I enjoy that." |
|  | Unconscious or habit | In addition to the physical and mental relief, some sedentary maintenance situations are unconscious or habitual. Since most patients do not simply sit, but perform other activities while sitting for a long time, their attention is often on other activities. | N13: "The longest time I sit is to check WeChat."  N5: "When I watch TV, sometimes I get so hooked that I just keep watching." |
|  | Tasks urgently needing completion | Urgent task completion is also a common scenario for sedentary maintenance, and older people with T2DM will continue to sit for long periods of time when a given task is not completed. | N5: "Once you start, each episode makes you want to keep watching continuously."  N13: "You just think, 'I'll push through and finish reading this article.'" |
|  | No sedentary interference items in the social environment for success | In the immersive maintenance situation of sedentary behavior, the social distraction-free victory items were the same as in the trigger situation. Non-competitive behavior and failure of competitive behavior also lead to the occurrence of sedentary maintenance situations. | N4: "I just lie down and watch TV." |
|  | Material environment facilitates | In the immersive maintenance situation of sedentary behavior, the physical environment contributes to and triggers the same situation. The sedentary maintenance situation occurs due to external environmental factors. | N1: "I basically don't use the computer anymore, but I still check my iPad and phone. Actually, I don’t look at them for long periods, but once I start, I can keep going for a long time."  N10: "Sometimes, once I start, I’ll be on it for hours."  N22: "When playing mahjong, I usually sit for three or four hours. When watching TV, I often lie on the sofa. Sometimes, I can also spend a long time scrolling through my phone." |
| Sedentary transfer breaks the situation | Changes in physical comfort | The term "transferential" is used to describe sedentary breaking situations because most sedentary behavior breaks with other behaviors. For example, get up to go to the toilet, get up to do activities, get up to drink water, get up to buy food and cook. | N1: "The changes in my body are that I get tired easily—my butt hurts from sitting, my waist hurts, and my neck feels stiff."  N15: "Sitting for a long time definitely makes you tired. You see, when I sit, I don’t sit still the whole time. Like when I'm looking at my phone, I switch between sitting like this and sitting like that, changing positions. You see, this is how I do it."  N19: "I just get up and walk when I get tired of looking at the computer."  N20: "When I get really tired and can’t take it anymore, I’ll walk around for a bit, just to move around." |
|  | Development of negative mental states | Some patients mentioned that when sitting for a long time, there will be negative emotions such as boredom, irritability, confusion, depression, psychological exhaustion. | N22: "Psychologically, I feel tired after sitting for a long time. There’s this sense of fatigue that sometimes I can't even explain. It’s strange—sometimes, because I’m tired, I prefer to sit, but sitting for too long also makes me tired. So, everything needs to be in moderation." |
|  | Unconscious or habit | In addition to the physical and mental changes that break up sedentary behavior, some behaviors are unconscious and habitual. | N1: "I don’t really feel bad emotions like frustration or fatigue after sitting for a long time. Those dark moods aren't really noticeable. I’m quite an optimistic person."  N4: "There’s no definite rule for this." |
|  | Disturbances in the social environment affecting successful outcomes emerge | In addition to physical and mental changes and unconsciousness or habit, sedentary behavior can also be broken when society appears to interfere with winning. At this point, the basic condition is that there are winning items (shopping, working, dating, etc.) and compete to win. | N22: "Nothing much, I usually just sit and watch TV. Otherwise, it’s either time to use the bathroom, cook, or if I get tired of sitting, I’ll get up and move around a bit."  N23: "Well... maybe when it's time to go to the bathroom or buy groceries. If it’s time to buy groceries, or sometimes I’ll just play with my phone. If my neck gets tired... when I’m feeling tired, I might get up and walk around if I’m uncomfortable." |
|  | Awareness of self-correction | It is also possible to break up sitting when older people with T2DM become self-correcting. | N1: "I just feel that sitting for too long isn’t good, so I get up to move around."  N13: "First of all, I have this understanding in my mind..." |
|  | Material environment is favorable | When sedentary behavior is broken, older T2DM patients refer to a range of items such as toilets, cups, kitchens, meals, rags, etc. When these items are present, the physical environment is favorable for breaking up sedentary behavior. | N6: "When I'm sitting, I know I need to get up after a while—go to the bathroom, or go to the kitchen to drink some water... I don't even use a thermos anymore. I just use an electric heating pad to keep water at 45°C, and I’ll go over and drink a couple of sips, then come back. I do it consciously to make myself move around... I’ve been doing this for many years. After one episode of a TV show ends, I quickly go to the kitchen to do something, like using the bathroom or washing something, then come back for the next episode." |
