# Supplementary material for: Telehealth Intervention to Reduce Sedentary Behavior in Older Adults With Type 2 Diabetes: Development and Feasibility Study
Source: J Med Internet Res. 2026 Mar 26;28:e80827. doi: 10.2196/80827 (PMC13020683; doi:10.2196/80827)
Supplement: Multimedia Appendix 6 [file jmir-v28-e80827-s006.docx]

**Appendix 6：Intervention function APEASE score**

| **Determinants** | **Potential**  **Intervention function** | **APEASE Criteria *M*（*SD*）** | | | | | | |
| --- | --- | --- | --- | --- | --- | --- | --- | --- |
|  |  | **Acceptability** | **Practicability** | **Effectiveness/**  **cost-effectiveness** | **Affordability** | **Side effects/**  **safety** | **Equity** | **Total Score** |
| Knowledge | Education | 2.84（0.37） | 2.95（0.23） | 2.26（0.73） | 2.95（0.23） | 3.00（0.00） | 2.89（0.32） | 16.89（1.10） |
| Intentions | Education | 2.74（0.56） | 2.05（0.85） | 1.95（0.52） | 2.37（0.50） | 3.00（0.00） | 2.79（0.42） | 14.89（1.37） |
|  | Persuasion | 2.63（0.68） | 2.16（0.76） | 1.89（0.66） | 2.37（0.60） | 3.00（0.00） | 2.32（0.89） | 14.37（1.80） |
|  | Incentivization | 2.95（0.23） | 2.63（0.50） | 2.26（0.45） | 2.26（0.45） | 2.74（0.45） | 2.37（0.50） | 15.21（1.08） |
|  | Coercion | 1.68（0.74） | 1.58（0.69） | 1.53（0.61） | 1.84（0.76） | 2.00（0.67） | 1.37（0.50） | 10.00（2.49） |
|  | Modelling | 1.53（0.51） | 1.68（0.58） | 2.32（0.75） | 1.89（0.57） | 1.63（0.83） | 1.63（0.60） | 10.68（1.73） |
| Social Support | Restriction | 2.11（0.74） | 1.74（0.65） | 2.26（0.65） | 2.11（0.74） | 1.63（0.60） | 1.63（0.76） | 11.47（1.90） |
|  | Environmental restructuring | 2.47（0.70） | 1.47（0.70） | 2.32（0.67） | 2.63（0.60） | 1.58（0.69） | 1.68（0.58） | 12.16（2.14） |
|  | Modelling | 2.68（0.48） | 1.53（0.51） | 2.00（0.58） | 1.68（0.58） | 1.84（0.60） | 1.68（0.48） | 11.42（1.35） |
|  | Enablement | 2.89（0.32） | 2.21（0.63） | 2.37（0.50） | 2.58（0.51） | 2.53（0.51） | 2.53（0.51） | 15.10（1.33） |

Notes：*M* = Mean，*SD* = Standard deviation
