# Supplementary material for: Telehealth Intervention to Reduce Sedentary Behavior in Older Adults With Type 2 Diabetes: Development and Feasibility Study
Source: J Med Internet Res. 2026 Mar 26;28:e80827. doi: 10.2196/80827 (PMC13020683; doi:10.2196/80827)
Supplement: Multimedia Appendix 7 [file jmir-v28-e80827-s007.docx]

**Appendix 7： The practical application of intervention strategies converts results**

| **Change Objectives** | **Performance Objectives** | **Intervention Functions** | **Policy Categories** | **Behavior Change Techniques** | **Content** | **Delivery Channels** | **Delivery Methods** |
| --- | --- | --- | --- | --- | --- | --- | --- |
| **Knowledge** |  |  |  |  |  |  |  |
| **The patient is aware of the hazards of sedentary behavior (incidence, influencing factors, consequences, etc.)** | PO.1 | Education | Service provision | 4.1 Instructions on how to perform the behavior  5.1 Information about health consequences  5.2 Significance of the consequences  6.1 behavior demonstration  6.2 Social comparison  9.1 Credible sources  13.2 Framing/restructuring | **Category 1: What is the relationship between sedentary behavior and Type 2 Diabetes?**   1. **What is Type 2 Diabetes?** 2. **What is the current epidemiological situation of diabetes?** 3. **What are the dangers of diabetes?** 4. **What is tertiary prevention for Type 2 Diabetes?** 5. **What is the relationship between sedentary behavior and Type 2 Diabetes?** 6. **What is the difference between sedentary behavior and physical inactivity?** 7. **What is the current situation of sedentary behavior among elderly diabetes patients?** 8. **What is the traditional Chinese medicine theoretical basis for sedentary behavior?** 9. **What are the dangers of sedentary behavior?** 10. **What does the World Health Organization say about sedentary behavior in elderly diabetes patients?** 11. **How long do I have to sit for it to be considered sedentary?** 12. **How can I calculate my sedentary time?** | Health Education Handbook  MG Animation Library  SMS Library | Health Education Handbook: At the start of implementation, researchers will send an electronic version to patients and their families. MG Animation Library: Every Tuesday and Friday, researchers will send one MG animation to the WeChat group, for a total of 1.5 months. SMS Library: Every Monday, Wednesday, Saturday, and Sunday, researchers will send text messages to the WeChat group (1-2 messages per day). |
| **The patient is aware of strategies to counteract sedentary behavior, such as reminders and behavior reversal** | PO.2 | Education | Service provision | **1.1 Goal setting (behavior)**  **1.2 Problem solving**  **1.4 Action planning**  **1.5 Review behavior goals**  **1.6 Current behavior and goal discrepancies**  **2.2 behavioral feedback**  **2.3 behavioral self-monitoring**  **3.1 Social support (unspecified)**  **3.2 Social support (practical)**  **4.1 Instructions on how to perform the behavior**  **5.2 Significance of consequences**  **5.4 Monitoring of emotional consequences**  **6.1 behavior demonstration**  **7.1 Cues/Reminders**  **8.2 behavior substitution**  **8.3 Habit formation**  **8.4 Habit reversal**  **8.7 Hierarchical tasks**  **10.3 Non-specific rewards**  **10.6 Non-specific incentives**  **10.7 Self-motivation**  **11.2 Reducing negative emotions**  **12.1 Reconstructing the physical environment**  **12.5 Adding cues to the environment**  **15.3 Reflecting on past successes** | **Category 2: How should sedentary behavior be addressed?**   1. **General strategies for countering sedentary behavior** 2. **Scenario 1: Strategies for countering sedentary behavior while using a smartphone** 3. **Scenario 2: Strategies for countering sedentary behavior while watching TV** 4. **Scenario 3: Strategies for countering sedentary behavior while using a computer** 5. **Scenario 4: Strategies for countering sedentary behavior while reading** 6. **Scenario 5: Strategies for countering sedentary behavior during transportation** 7. **Scenario 6: Strategies for countering sedentary behavior during hobbies** (Note: Adjusted numbering for consistency) 8. **Scenario 7: Strategies for countering sedentary behavior during social activities** |  |  |
| **Family members are aware of the current status, influencing factors, and consequences of sedentary behavior** | PO.3 | Education | Service provision | **4.1 Instructions on how to perform the behavior**  **5.1 Information about health consequences**  **5.2 Significance of consequences**  **6.1 behavior demonstration**  **6.2 Social comparison**  **9.1 Credible sources**  **13.2 Framing/restructuring** | Same as PO.1，as follows：  **Category 1: What is the relationship between sedentary behavior and Type 2 Diabetes?**   1. **What is Type 2 Diabetes?** 2. **What is the current epidemiological situation of diabetes?** 3. **What are the dangers of diabetes?** 4. **What is tertiary prevention for Type 2 Diabetes?** 5. **What is the relationship between sedentary behavior and Type 2 Diabetes?** 6. **What is the difference between sedentary behavior and physical inactivity?** 7. **What is the current situation of sedentary behavior among elderly diabetes patients?** 8. **What is the traditional Chinese medicine theoretical basis for sedentary behavior?** 9. **What are the dangers of sedentary behavior?** 10. **What does the World Health Organization say about sedentary behavior in elderly diabetes patients?** 11. **How long do I have to sit for it to be considered sedentary?** 12. **How can I calculate my sedentary time?** |  |  |
| **The patient is aware of how to engage in physical activity to alleviate discomfort from prolonged sitting** | PO.4 | Education | Service provision | 1.4 Action planning  4.1 Instructions on how to perform the behavior  5.1 Information about health consequences  5.2 Significance of consequences  6.1 behavior demonstration  8.2 behavior substitution  8.3 Habit formation  8.4 Habit reversal  8.7 Graded tasks  9.1 Credible sources | **Category 3: How to alleviate discomfort from sedentary behavior?**   1. **How to choose the right chair?** 2. **What is the correct sitting posture?** 3. **Which acupressure points can alleviate discomfort from prolonged sitting?**   **Category 4: How should one engage in physical activity?**   1. **What are the benefits of physical activity?** 2. **What does the diabetes physical activity guideline say?** 3. **What types of exercise are recommended?** 4. **What is Traditional Chinese Medicine wellness guidance technique?** 5. **What should be considered when engaging in physical activity?** 6. **What are the best times for physical activity in diabetes management?** 7. **Who should not engage in physical activity?** |  |  |
| **Social Support** |  |  |  |  |  |  |  |
| **Researchers/family members provide social support to the patient regarding 'awareness of the risks and consequences of sedentary behavior'**. | PO.1 | Enablement | Service provision | **1.2 Problem Solving**  **3.1 Social Support (unspecified)**  **3.2 Social Support (practical)**  **3.3 Social Support (emotional)**  **12.2 Reconstructing the Social Environment**  **15.1 Verbal Persuasion about Ability** | **Researchers/family members provide patients with information, resources, and emotional support:**  **Information:** What is sedentary behavior? What is the current status of sedentary behavior? What factors influence sedentary behavior? What are the consequences of sedentary behavior?  **Resources:** Ways to access relevant knowledge  **Emotional:** Regularly addressing questions and concerns | WeChat group | **WeChat Q&A group:** Every Friday from 14:00 to 15:00, researchers will answer patients' questions and resolve any doubts, encouraging patients to interact and share experiences in the group. |
| **Researchers/family members provide social support to the patient regarding 'choosing appropriate strategies to cope with sedentary behavior'**. | PO.2 | Enablement | Service provision | **1.2 Problem Solving**  **3.1 Social Support (unspecified)**  **3.2 Social Support (practical)**  **3.3 Social Support (emotional)**  **12.2 Reconstructing the Social Environment**  **15.1 Verbal Persuasion about Ability** | **Researchers/family members provide patients with information, resources, and emotional support:**  **Information:** What are sedentary behavior coping strategies? What kinds of coping strategies are there for sedentary behavior?  **Resources:** Ways to access relevant knowledge  **Emotional:** Regularly addressing questions and concerns |  |  |
| **Researchers/family members provide social support to the patient regarding 'reminders and interruptions during prolonged sitting'**. | PO.3 | Enablement | Service provision | **1.2 Problem Solving**  **3.1 Social Support (unspecified)**  **3.2 Social Support (practical)**  **3.3 Social Support (emotional)**  **12.2 Reconstructing the Social Environment**  **15.1 Verbal Persuasion about Ability** | **Researchers/family members provide patients with information, resources, and emotional support:**  **Information:** When to interrupt? How to interrupt?  **Resources:** Ways to access relevant knowledge  **Emotional:** Regularly addressing questions and concerns |  |  |
| **Researchers/family members provide social support to the patient regarding 'engaging in appropriate physical activities to alleviate discomfort from prolonged sitting'**. | PO.4 | Enablement | Service provision | **1.2 Problem Solving**  **3.1 Social Support (unspecified)**  **3.2 Social Support (practical)**  **3.3 Social Support (emotional)**  **12.2 Reconstructing the Social Environment**  **15.1 Verbal Persuasion about Ability** | **Researchers/family members provide patients with information, resources, and emotional support:**  **Information:** What is physical activity? The importance of physical activity, what factors influence physical activity? How to engage in physical activity?  **Resources:** Ways to access relevant knowledge  **Emotional:** Regularly addressing questions and concerns |  |  |
| ****Intentions**** |  |  |  |  |  |  |  |
| The patient expresses an intention to reduce sedentary behavior. | PO.1 | Incentivization | Service provision | **2.2 behavioral Feedback**  **7.2 Prompt signal reward**  **10.1 Material incentive (behavior)** | Inform patients that upon reaching the anticipated goals or at the end of the study, material incentives will be provided, including a health education manual on sedentary behavior, a guide for high-risk diabetic foot management, a comprehensive self-management guide for Type 2 diabetes patients, an illustrated manual of exercises for diabetes, and occasional WeChat red envelopes (cash gifts). | **Material Incentives Package:** Includes a health education manual on sedentary behavior, a guide for high-risk diabetic foot management, a comprehensive self-management guide for Type 2 diabetes patients, an illustrated manual of exercises for diabetes, and occasional WeChat red envelopes (cash gifts).**SMS Library:** Provides prompt signal rewards. | **Material Incentives Package:** Upon reaching the anticipated goals or at the conclusion of the research, researchers will distribute incentive materials. |
| The patient expresses an intention to adopt appropriate coping strategies. | PO.2 | Incentivization | Service provision | **2.2 behavioral Feedback**  **7.2 Prompt signal reward**  **10.1 Material incentive (behavior)** | Inform patients that upon reaching the anticipated goals or at the end of the study, material incentives will be provided, including a health education manual on sedentary behavior, a guide for high-risk diabetic foot management, a comprehensive self-management guide for Type 2 diabetes patients, an illustrated manual of exercises for diabetes, and occasional WeChat red envelopes (cash gifts). |  |  |
| Family members express an intention to monitor and remind the patient. | PO.3 | Incentivization | Service provision | **2.2 behavioral Feedback**  **7.2 Prompt signal reward**  **10.1 Material incentive (behavior)** | Inform patients that upon reaching the anticipated goals or at the end of the study, material incentives will be provided, including a health education manual on sedentary behavior, a guide for high-risk diabetic foot management, a comprehensive self-management guide for Type 2 diabetes patients, an illustrated manual of exercises for diabetes, and occasional WeChat red envelopes (cash gifts). |  |  |
| The patient expresses an intention to engage in physical activity. | PO.4 | Incentivization | Service provision | **2.2 behavioral Feedback**  **7.2 Prompt signal reward**  **10.1 Material incentive (behavior)** | Inform patients that upon reaching the anticipated goals or at the end of the study, material incentives will be provided, including a health education manual on sedentary behavior, a guide for high-risk diabetic foot management, a comprehensive self-management guide for Type 2 diabetes patients, an illustrated manual of exercises for diabetes, and occasional WeChat red envelopes (cash gifts). |  |  |
