# Supplementary material for: Telehealth Intervention to Reduce Sedentary Behavior in Older Adults With Type 2 Diabetes: Development and Feasibility Study
Source: J Med Internet Res. 2026 Mar 26;28:e80827. doi: 10.2196/80827 (PMC13020683; doi:10.2196/80827)
Supplement: Multimedia Appendix 8 [file jmir-v28-e80827-s008.docx]

Appendix 8："Double-S" intervention strategy for elderly patients with type 2 diabetes

| **Item** | **Item Content** | **This Study** |
| --- | --- | --- |
|  | - ****Abbreviation**** |  |
| 1. | A name or phrase used to describe the intervention strategy. | **"Double-S Plan" for Community-Dwelling Elderly Patients with Type 2 Diabetes** Note: **Stop sedentary behavior, Stand up** |
|  | - Why |  |
| 2. | Describe the basic rationale, theory, or objectives behind the essential components of the intervention strategy. | **Intervention Matching、Behavior Change Wheel、Jorge's Conceptual Model of Sedentary Behavior in Older Adults** |
|  | - ****Research Content**** |  |
| 3. | **Research Content (Materials):** Describe any physical objects or related informational materials used in the intervention strategy, including those used during the delivery of the intervention to participants or those used to train the intervention providers. Provide information on how to access or obtain these materials. | **Electronic Health Education Manual**  ****MG Animation Library****  ****Text Message Library****  ****WeChat Q&A Group****  ****Material Incentive Package**** |
| 4. | **Operational Process:** Describe each procedure, activity, and/or process involved in the implementation of the intervention strategy, including any facilitative or supportive activities. | **Electronic Health Education Manual:** At the beginning of the implementation, the researcher sends an electronic version to the patients and their families.  ****MG Animation Library**:** Every Tuesday and Friday, the researcher sends an MG animation to the WeChat group.  **Text Message Library:** Every Monday, Wednesday, Saturday, and Sunday, the researcher sends SMS messages to the WeChat group (1-2 messages per day).  ****WeChat Q&A Group**:** Every Friday from 14:00 to 15:00, the researcher answers questions for patients and encourages communication within the group.  ****Material Incentive Package**:** When patients reach the expected goals or at the end of the study, the researcher distributes incentive materials. |
|  | - **Intervention Implementers** |  |
| 5. | For Different Types of Intervention Implementers (e.g., Psychotherapists, Assistant Nurses), describe their expertise, background, and professional training received. | All are implemented by the researcher. |
|  | - **Operational Method** | WeChat Group |
| 6. | Describe the operational method of the intervention strategy (whether it is face-to-face or through other means, such as via the internet or phone), and whether the intervention strategy is conducted individually or in groups. |  |
|  | - **Implementation Location** |  |
| 7. | Describe the type of location where the intervention strategy is implemented, including necessary infrastructure or relevant features. | It is mainly implemented through WeChat groups. |
|  | - **Implementation Time and Intensity** |  |
| 8. | Describe the frequency and time periods of the intervention strategy, including the number of treatment sessions, scheduling, duration, intensity, or dosage. | **Electronic Health Education Manual:** At the beginning of the implementation, the researcher sends an electronic version to the patients and their families.  ****MG Animation Library**:** Every Tuesday and Friday, the researcher sends an MG animation to the WeChat group.  ****Text Message Library****: Every Monday, Wednesday, Saturday, and Sunday, the researcher sends SMS messages to the WeChat group (1-2 messages per day).  ****WeChat Q&A Group**:** Every Friday from 14:00 to 15:00, the researcher answers questions for patients and encourages communication within the group.  ****Material Incentive Package**:** When patients reach the expected goals or at the end of the study, the researcher distributes incentive materials. |
|  | - **Personalized Plan** |  |
| 9. | If the intervention strategy requires individual adjustments, its content, reasons, timing, and methods should be described. | The intervention is urgent to implement; the personalized plan may include personalized problem-solving, goal setting, etc. |
|  | - **Plan Modification** |  |
| 10. | If modifications were made to the intervention strategy during the study, please describe what was changed, why the modification was made, when it was made, and how it was modified. | The intervention is urgently needed to be implemented. |
|  | - **Effect** |  |
| 11. | Expected Effect: If the adherence or consistency of the intervention strategy has been evaluated, please describe how it was assessed, who conducted the assessment, and if any strategies were used to maintain or improve consistency, please describe these strategies. | The intervention is urgently needed to be implemented. |
| 12. | Expected Effect: If the adherence or consistency of the intervention strategy has been evaluated, please describe how it was assessed, who conducted the assessment, and if any strategies were used to maintain or improve consistency, please describe these strategies. | The intervention is urgently needed to be implemented. |
