# Supplementary material for: Telehealth Intervention to Reduce Sedentary Behavior in Older Adults With Type 2 Diabetes: Development and Feasibility Study
Source: J Med Internet Res. 2026 Mar 26;28:e80827. doi: 10.2196/80827 (PMC13020683; doi:10.2196/80827)
Supplement: Multimedia Appendix 9 [file jmir-v28-e80827-s009.docx]

Appendix 9：**"Double-S" program APEASE score results**

| **Determinants** | **Components** | **APEASE Criteria *M*（*SD*）** | | | | | | |
| --- | --- | --- | --- | --- | --- | --- | --- | --- |
|  |  | **Acceptability** | **Practicability** | **Effectiveness/**  **cost-effectiveness** | **Affordability** | **Side-effecst/safety** | **Equity** | **Total Score** |
| Knowledge | 1. Electronic Health Education Manual | 3.00（0.00） | 3.00（0.00） | 3.00（0.00） | 3.00（0.00） | 3.00（0.00） | 3.00（0.00） | 18.00（0.00） |
|  | 1. MG Animation Library | 3.00（0.00） | 3.00（0.00） | 3.00（0.00） | 3.00（0.00） | 3.00（0.00） | 3.00（0.00） | 18.00（0.00） |
|  | 1. Text Message Library | 3.00（0.00） | 3.00（0.00） | 3.00（0.00） | 3.00（0.00） | 3.00（0.00） | 3.00（0.00） | 18.00（0.00） |
| Social Support | 1. WeChat Q&A Group | 2.88（0.35） | 2.88（0.35） | 2.88（0.35） | 2.88（0.35） | 2.88（0.35） | 2.88（0.35） | 17.25（2.12） |
| Intentions | 1. Material Incentive Package | 3.00（0.00） | 3.00（0.00） | 3.00（0.00） | 3.00（0.00） | 3.00（0.00） | 3.00（0.00） | 18.00（0.00） |
