# Supplementary material for: Telehealth Intervention to Reduce Sedentary Behavior in Older Adults With Type 2 Diabetes: Development and Feasibility Study
Source: J Med Internet Res. 2026 Mar 26;28:e80827. doi: 10.2196/80827 (PMC13020683; doi:10.2196/80827)
Supplement: Multimedia Appendix 10 [file jmir-v28-e80827-s010.docx]

Appendix 10：**The five components of the "Double-S" Plan**

**"Double-S" Plan**, hereinafter referred to as the "Double-S Plan," is an abbreviation for **Stop Sedentary behavior, Stand Up**, developed based on intervention matching, the behavior Change Wheel, and Jorge's Conceptual Model of Sedentary behavior in Older Adults. The **"Double-S" Plan** includes five major components:

- Electronic Health Education Manual
- MG Animation Library
- Text Message Library
- WeChat Q&A Group
- Material Incentive Package

| **Components** | **Comntext** | **Partial content example** |
| --- | --- | --- |
| 1. Electronic Health Education Manual | The electronic health education manual includes 4 major categories and 29 topics | 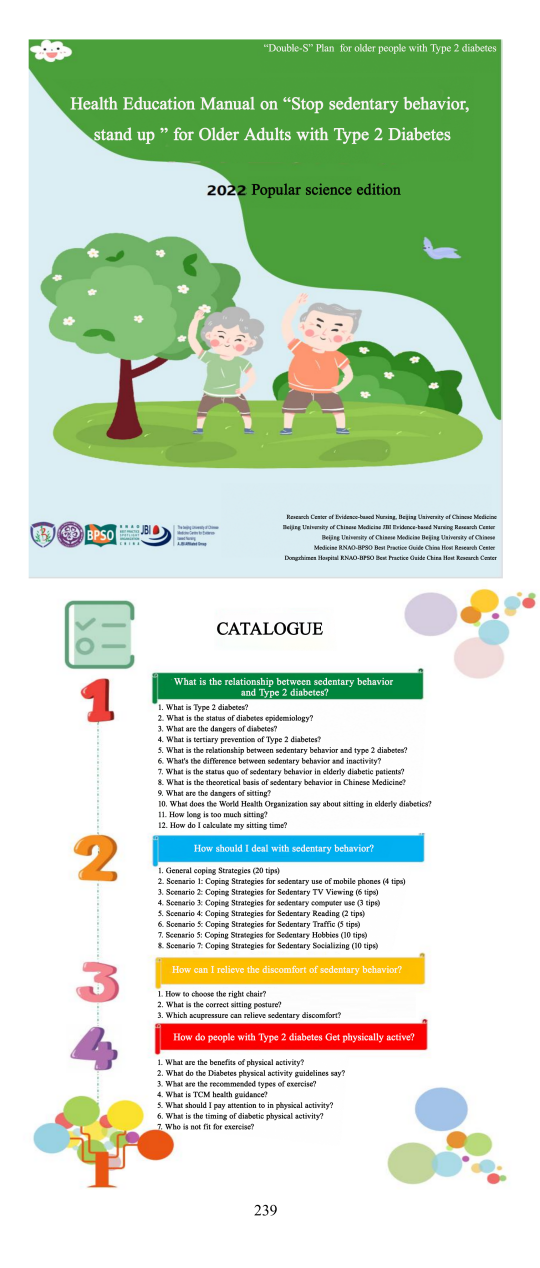 |
| 1. MG Animation Library | The content of the MG Animation Library also revolves around these 4 major categories and 29 topics. Ultimately, the MG Animation Library includes 14 self-produced videos and 8 referenced videos. | 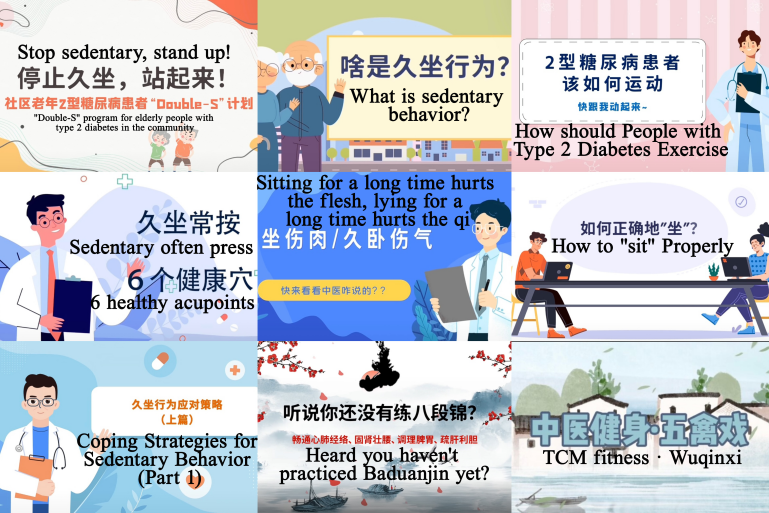 |
| 1. Text message library | The content of the text message library also revolves around these 4 major categories and 29 topics. The text message library includes 138 messages. | 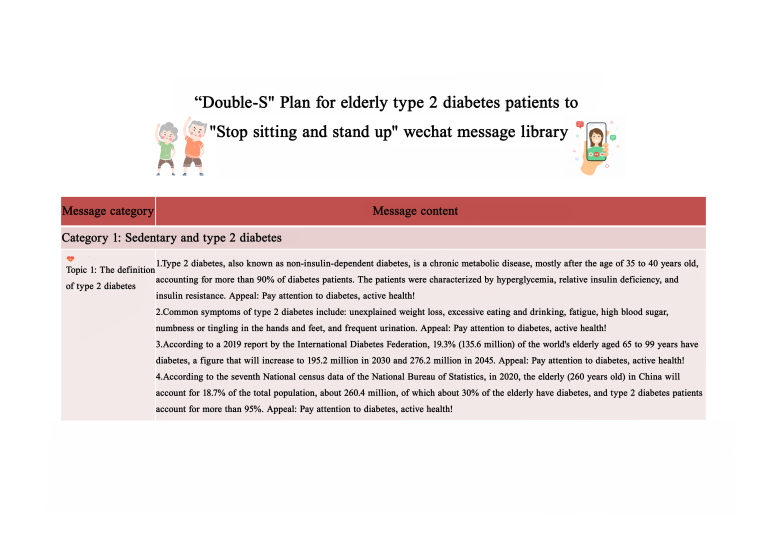 |
| 1. WeChat Q&A group | The WeChat Q&A Group is held every Friday from 14:00 to 15:00, where the researcher answers questions for patients and encourages communication within the group. The WeChat group is also an important channel for obtaining information and provides primary social support. | 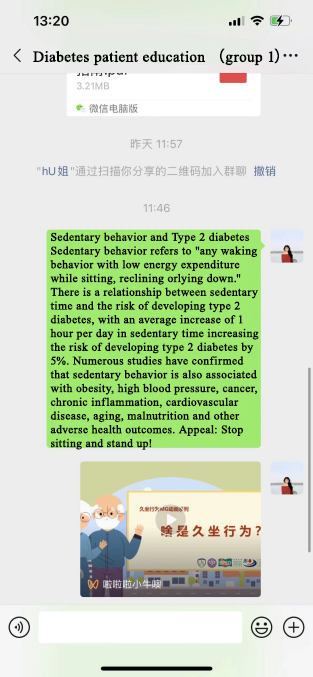 |
| 1. Material Incentive Package | **Material Incentives Package:** Includes a health education manual on sedentary behavior, a guide for high-risk diabetic foot management, a comprehensive self-management guide for Type 2 diabetes patients, an illustrated manual of exercises for diabetes, and occasional WeChat red envelopes (cash gifts). |  |
