# Supplementary material for: Telehealth Intervention to Reduce Sedentary Behavior in Older Adults With Type 2 Diabetes: Development and Feasibility Study
Source: J Med Internet Res. 2026 Mar 26;28:e80827. doi: 10.2196/80827 (PMC13020683; doi:10.2196/80827)
Supplement: Multimedia Appendix 11 [file jmir-v28-e80827-s011.docx]

Appendix 11：**General patient information (n=31)**

| Item | Category | n（%）/ $\bar{x}\pm s$ |
| --- | --- | --- |
| Year | — | 66.35 ± 3.94 |
| Gender | Male | 16（51.61） |
|  | Female | 15（48.39） |
| BMI（kg/m2） | 18.5 ≤ BMI < 24 | 11（35.48） |
|  | 24 ≤ BMI < 28 | 19（61.29） |
|  | ≥ 28 | 1（3.23） |
| Degree of education | Primary school and below | 4（12.90） |
|  | Junior high school | 16（51.61） |
|  | Senior high school/  Technical secondary school | 8（25.81） |
|  | Junior college and above | 3（9.68） |
| Marital status | Married | 27（87.10） |
|  | Others | 4（12.90） |
| Residence mode | Living alone | 2（6.45） |
|  | Not living alone | 29（93.55） |
| Income | ≤ 1999 | 4（12.90） |
|  | 2000~3999 | 15（48.39） |
|  | 4000~5999 | 9（29.03） |
|  | ≥ 6000 | 3（9.68） |
| Combining chronic disease | Yes | 22（70.97） |
|  | No | 9（29.03） |
| Course of the disease（year） | — | 16.87 ± 5.33 |
| Fasting blood glucose | — | 7.08 ± 0.85 |
| Systolic blood pressure（mmHg） |  | 126.29 ± 13.15 |
| Diastolic blood pressure（mmHg） |  | 63.68 ± 8.11 |
| Sedentary behavior（ ≥ 6 h/d） | Yes | 29（93.55） |
|  | No | 2（6.45） |
